# Muscle weakness, pain, and fatigue impair daily function in chronic kidney disease: a cross-sectional analysis from the I-RACE study

**DOI:** 10.1080/0886022X.2026.2637300

**Published:** 2026-03-04

**Authors:** Roseanne E. Billany, Heitor S. Ribeiro, Courtney J. Lightfoot, Thomas J. Wilkinson, Alice C. Smith, Matthew P. M. Graham-Brown, Emma L. Watson

**Affiliations:** a Division of Cardiovascular Sciences, University of Leicester, Leicester, UK; b Leicester Partnership for Kidney Health Research, University of Leicester, Leicester, UK; c NIHR Leicester Biomedical Research Centre, University of Leicester and University Hospitals of Leicester NHS Trust, UK; d University of Brasília, Faculty of Health Sciences, Brasília, Federal District, Brazil; e Leicester Diabetes Centre, University Hospitals of Leicester NHS Trust, Leicester, UK; f Division of Global, Lifestyle and Metabolic Health, University of Leicester, Leicester, UK

**Keywords:** Neuromuscular manifestations, musculoskeletal pain, dialysis, kidney failure

## Abstract

Muscle dysfunction symptoms such as weakness and cramps are common in people with chronic kidney disease (CKD), but their impact on activities of daily living (ADLs) is not well understood. This study explored the association between muscle symptoms and patient-reported ADL impact in individuals with and without CKD. This cross-sectional secondary analysis of the I-RACE study (ISRCTN11596292) included adults (≥18 years) across the CKD spectrum and adults without CKD. Participants completed a bespoke Muscle Symptoms Scale assessing muscle-related symptoms and their impact on ADLs (daily activities, socializing, working, exercising). Group differences were tested using univariate general linear models; associations were examined *via* linear regression. Among 1048 participants (304 non-CKD, 345 non-dialysis CKD, 281 dialysis, 118 transplant), mean age was 57 ± 17 years and 51% were female. Muscle symptoms (weakness, tiredness, aches/pains, cramps/tightness, reduced muscle size, restless legs) were significantly worse in CKD groups (all *p* < 0.05). Dialysis and transplant recipients reported more severe symptoms than non-dialysis CKD, with dialysis patients reporting the worst weakness and muscle size reduction. ADL impact followed a similar pattern, with dialysis patients most affected. All muscle symptoms were significantly associated with ADL impact in CKD, particularly weakness and reduced muscle size (all *p* < 0.05). Muscle dysfunction symptoms are more severe in CKD, especially in kidney failure. These symptoms are strongly associated with impaired ADLs, highlighting muscle size and strength as potential targets for intervention through exercise.

## Introduction

Sarcopenia, characterized by a loss of muscle strength, mass and function is a frequent complication of chronic kidney disease (CKD) [1], affecting around 28% of people with advanced disease [2,3]. CKD is associated with muscle wasting and sarcopenia, even in earlier stages, through mechanisms related to inflammation, intramuscular fat, oxidative stress, and hormonal imbalances [4,5]. In addition, complications may extend to joints and connective tissues, causing higher symptoms of pain [6] and muscle dysfunction [7,8].

Fatigue, cramps, and weakness are common symptoms often attributed to muscle dysfunction in those living with CKD [9–12], but these symptoms may also be attributed to other features of CKD, such as anemia, electrolyte imbalances, and uremia [13]. Nevertheless, these symptoms likely contribute to reduced physical function, ability to engage in daily activities essential for independent living, to work, and to participate in social and sporting pursuits, potentially contributing to further physical decline [13]. Such activities underpin economic contribution to society as well as full and meaningful participation in individually valued aspects of life; indeed, patients and caregivers give the highest priority to fatigue and life participation as important outcomes in kidney care [14]. Muscle dysfunction and related symptoms may therefore ultimately be costly for health and social services as well as having significant negative effects on quality of life [15].

Investigating how symptoms of muscle dysfunction manifest for patients with CKD and how they impact daily activities is crucial for devising targeted, non-pharmacological, interventions (*e.g.* exercise, diet, and lifestyle behavior modification) that restore health and improve life participation [16]. To address this knowledge gap, we investigated the association between symptoms of muscle dysfunction and impact on activities of daily living (ADLs) in people with and without CKD.

## Materials and methods

### Study design

This was a cross-sectional study and a secondary analysis of the ‘Intramuscular and Inflammatory Response to Acute Exercise in Chronic Disease (I-RACE)’ study conducted between November 2017 and July 2020 (ISRCTN11596292). Further detailed materials and methods are published elsewhere [17]. In brief, participants were invited to complete a survey pack containing a series of questionnaires, including a bespoke Muscle Symptoms Scale (MSS), during medical appointments or through postal services. National ethical approval was granted by the East Midlands – Derby Research Ethics Committee (ref: 17/EM/0357). Written informed consent was provided by all participants, and the study adhered to the principles of the Declaration of Helsinki.

### Study sample and recruitment

People aged ≥18 years with a confirmed diagnosis of CKD across the spectrum of the disease, including non-dialysis dependent (NDD), hemodialysis (HD), peritoneal dialysis (PD), and kidney transplant recipients (KTRs) managed as outpatients at seven hospital sites across the United Kingdom were identified by their clinical teams and invited to participate in person or by post. Patients were categorized into three groups: i) NDD-CKD (CKD stages 1–2: ≥60 mL/min/1.73 m^2^; 3: 30–59 mL/min/1.73 m^2^; 4: 15–29 mL/min/1.73 m^2^; 5: <15 mL/min/1.73 m^2^); ii) dialysis (both HD and PD); iii) KTRs. With informed consent, clinical data were extracted from electronic medical records and estimated glomerular filtration rate (eGFR) calculated using the CKD-EPI equation. In addition, people without CKD (non-CKD group) were invited from hospital and university staff, their family and social contacts, and ‘significant others’ of patient participants. Clinical records were not accessed for the non-CKD group and all information was self-reported *via* the survey.

### Bespoke muscle symptoms scale

The term ‘muscle dysfunction symptoms’ in this study encompasses both perceptual or sensory symptoms (e.g. aches, fatigue) and physiological indicators of muscle impairment (e.g. weakness, perceived reduction in muscle size). To appropriately capture the breadth of symptoms related to muscle disease described by patients with CKD, we designed the bespoke Leicester Kidney Exercise Team muscle symptom scale (MSS; Supplementary Material 1). It comprises two domains and a total score: i) rating of six muscle-related symptoms (weakness, tiredness, aches or pains, cramps and tightness, muscle size reduction in the last 6 months, and restless leg syndrome [RLS] in the last 6 months); and ii) rating muscle-related symptom impact on four types of activity over the last 6 months (performing daily activities, socializing, working, and exercising).

Participants were asked to rate on a scale from 0 to 10 to indicate the extent to which they agree or disagree with each statement or question. If a symptom or problem was listed but not experienced by the participant, a score of zero was recorded. Muscle-related symptoms scores ranged from 0 to 60, muscle-related impact from 0 to 40, and the total score from 0 to 100. Higher scores indicate greater severity of muscle-related symptoms and their impact. Patients with missing data on the MSS were excluded from analysis.

### Statistical analysis

Normality of data was assessed using the Kolmogorov–Smirnov test. Normally distributed data are presented as mean ± SD, non-normally distributed data are presented as median (IQR). Frequencies are presented as number and percent (i.e. excluding missing data).

Differences of symptoms of muscle dysfunction across groups were determined using a univariate General Linear Model (GLM), adjusted for age, with Bonferroni post-hoc correction. Associations between symptoms of muscle dysfunction (independent) and impact on ADLs (dependent) were tested by using linear regression analyses (univariate and multivariable) adjusted for age, sex, and diabetes. In the multivariable analysis, all symptoms of muscle dysfunction were included in the model. All analyses were performed using the Statistical Package for the Social Sciences (version 29.0, SPSS Inc, Chicago, USA) and GraphPad Prism (version 8, GraphPad Software, San Diego, USA). A two-tailed *p*-value < 0.05 was considered statistically significant.

## Results

### Recruitment

**Figure 1.**
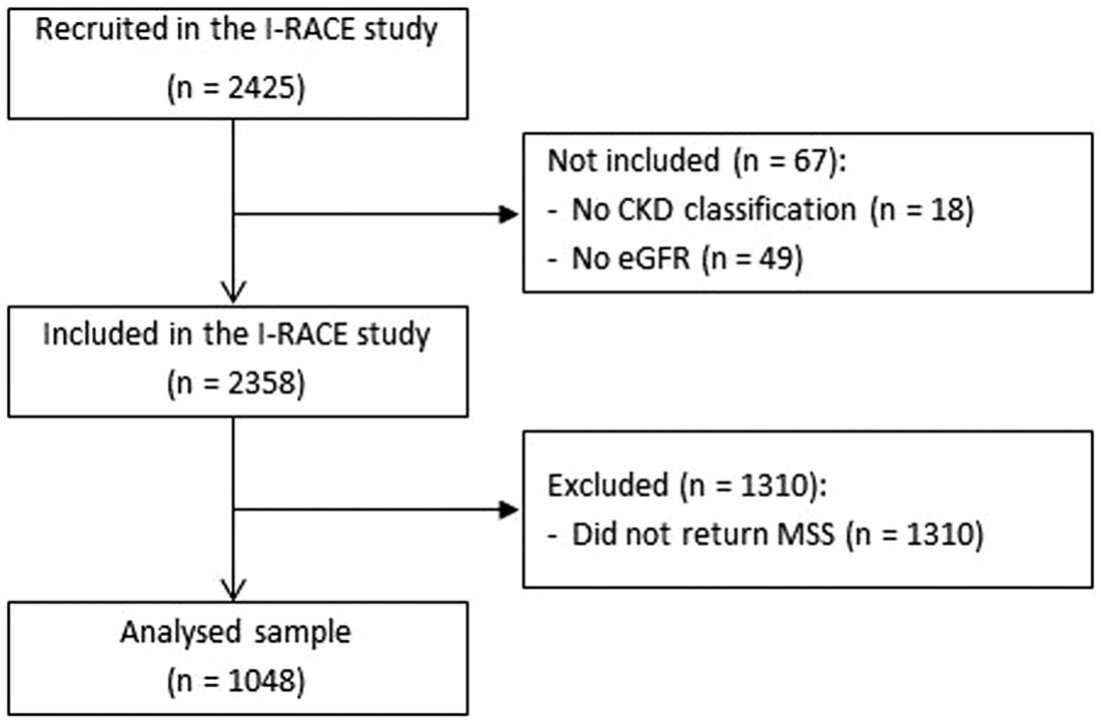
Flowchart of participants’ recruitment. CKD: chronic kidney disease; eGFR: estimated glomerular filtration rate; MSS: muscle symptom scale.

A total of 2425 participants were recruited to the I-RACE study. Of these, 18 did not have a CKD classification recorded, and 49 had no reported eGFR and were therefore excluded leaving a total of 2358 participants (Figure 1). Of these 2358, 1310 participants did not return a completed MSS, leaving 1048 participants included in the analysis.

### Characteristics of the analyzed sample

Characteristics of the 1048 participants with eligible MSS data included are shown in Table 1 stratified by CKD stages. Overall, participants were 57 ± 17 years old, 51% female, and 66% White British. Hypertension (67%) and diabetes (34%) were the most prevalent comorbidities in patients with CKD. Regarding NDD-CKD stages, 4% (*n* = 12) were stage 1, 8% (*n* = 28) were stage 2, 35% stage 3 (*n* = 117), 39% stage 4 (*n* = 128), and 14% stage 5 (*n* = 46). Fourteen NDD-CKD had missing eGFR data.

**Table 1.** Demographic and clinical characteristics of the participants.

	Non-CKD (*n* = 304)	All CKD (*n* = 744)	NDD-CKD (*n* = 345)	Dialysis (*n* = 281)	KTR (*n* = 118)
Age (years) median [IQR]	48 [32, 58]	62 [51, 73]	68 [54, 77]	61 [53, 72]	54 [43, 61]
≥ 65 years, n (%)	47 (16.0)	323 (43.4)	190 (55.2)	113 (40.5)	20 (16.9)
Female, n (%)	220 (72.4)	325 (43.6)	161 (46.7)	112 (39.9)	52 (44.1)
Ethnicity, n (%)					
White British	144 (51.6)	531 (71.4)	301 (87.5)	138 (49.5)	92 (78.6)
Southeast Asian	55 (19.7)	78 (10.5)	17 (4.9)	50 (17.9)	11 (9.4)
African	20 (7.2)	31 (4.2)	6 (1.7)	23 (8.2)	2 (1.7)
Other	60 (21.5)	100 (13.4)	20 (5.8)	68 (24.4)	12 (10.3)
Clinical, median [IQR]					
eGFR (ml/min/1.73 m^2^)	–	–	28 [19, 43]	–	42 [29, 57]
Haemoglobin (g/L)	–	110 [95, 123]	115 [87, 132]	107 [96, 115]	109 [82, 126]
Albumin (g/L)	–	42 [39, 44]	42 [39, 45]	41 [38, 43]	42 [39, 44]
Comorbidities, n (%)					
Diabetes	17 (5.6)	254 (34.2)	103 (29.9)	124 (44.1)	27 (22.9)
Hypertension	–	496 (66.7)	229 (66.4)	188 (66.9)	79 (66.9)
Cancer	14 (4.6)	78 (10.5)	44 (12.8)	27 (9.6)	7 (5.9)
CVD	15 (4.9)	96 (12.9)	16 (4.6)	73 (26.0)	7 (5.9)
Musculoskeletal	87 (28.6)	143 (19.2)	75 (21.7)	48 (17.1)	20 (16.9)
Neurological	–	28 (3.8)	13 (3.8)	11 (3.9)	4 (3.4)

CVD: cardiovascular disease; eGFR: estimated glomerular filtration rate; IQR: interquartile range; KTR: kidney transplant recipients; NDD: non-dialysis dependent.

### Symptoms of muscle-related dysfunction on the muscle symptom scale

**Figure 2.**
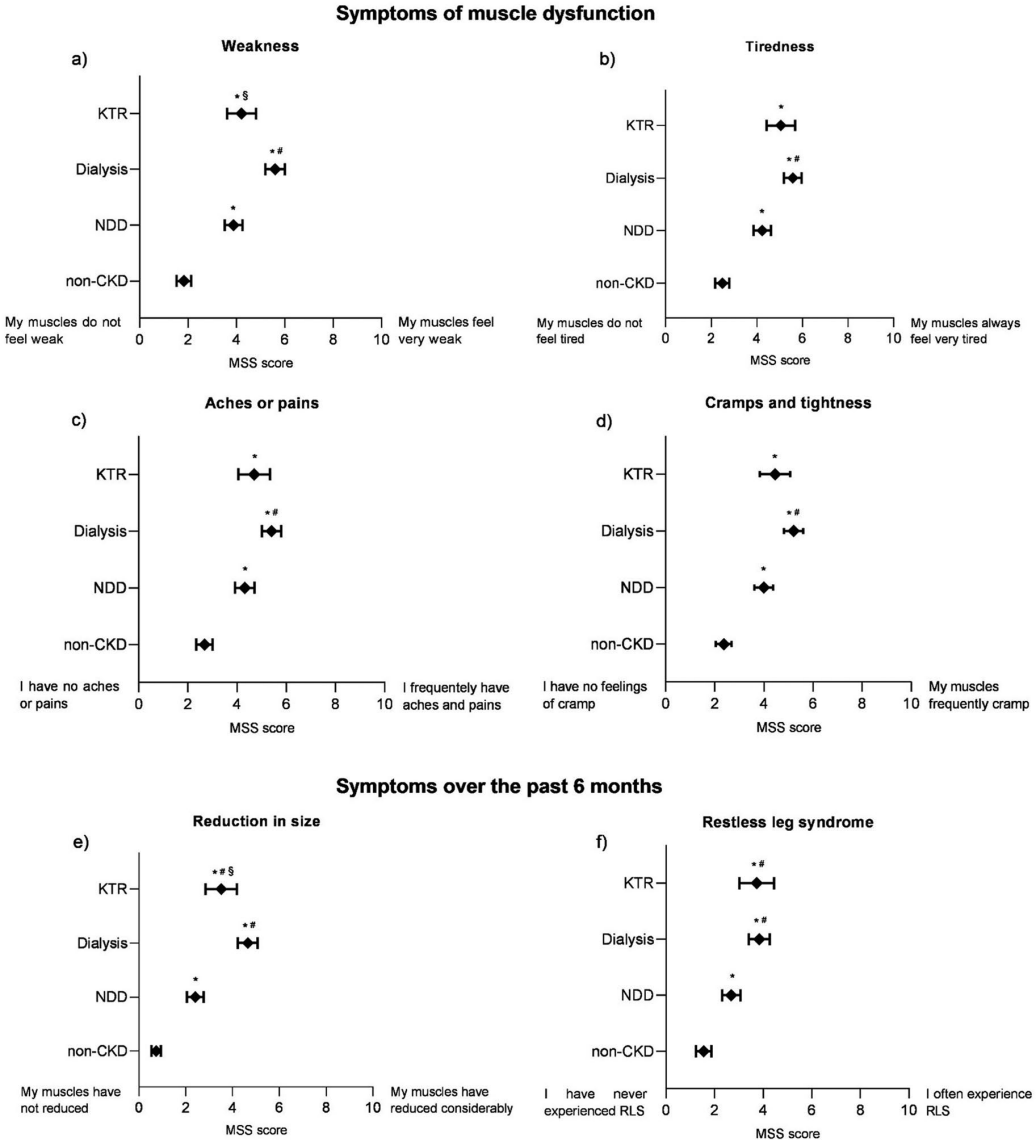
Symptoms of muscle dysfunction in people with and without CKD. a) weakness; b) tiredness; c) aches or pains; d) cramps and tightness; e) reduction in muscle size; f) restless leg syndrome. MSS score ranges from 0 to 10 to reflect the full response range of the scale. Data are displayed as mean and 95% confidence intervals. Non-CKD (*n* = 304); NDD (*n* = 345); Dialysis (*n* = 281); KTR (*n* = 118). CKD: chronic kidney disease; KTR: kidney transplant recipients; MSS: muscle symptom scale; NDD: non-dialysis dependent; RLS: restless leg syndrome. *Statistical difference to non-CKD; ^#^Statistical difference to non-dialysis dependent; ^§^Statistical difference to dialysis.

Symptoms of muscle-related dysfunction assessed by the MSS are shown in Figure 2 and numerical values in Supplementary Table S1. Participants without CKD had lower scores across the MSS than those with CKD for all symptoms (*p* < 0.05). Additionally, MSS scores were worse for people on dialysis compared to patients with NDD-CKD for all symptoms (*p* < 0.05), suggesting symptoms worsened with the progression to kidney failure. Significant differences between MSS scores for people on dialysis and KTR were observed for weakness and reduction in muscle size, which were worse in the dialysis group (*p* < 0.05).

### Impact of symptoms of muscle dysfunction on ADLs

**Figure 3.**
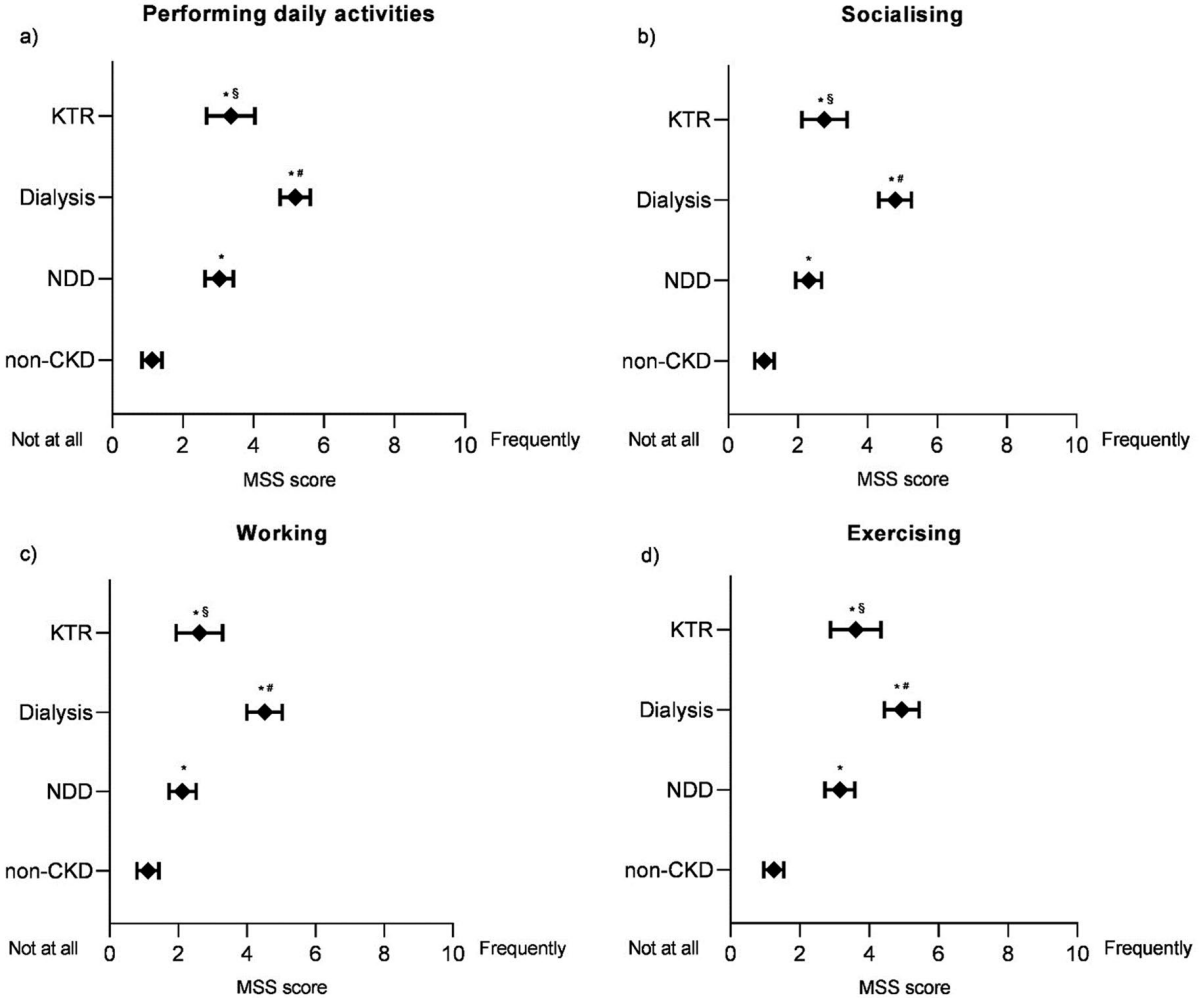
Impact of symptoms of muscle dysfunction over the past 6 months on ADLs. People answered for: ‘Over the past 6 months, my muscle symptoms have limited me from:’ a) performing daily activities; b) socializing; c) working; d) exercising. MSS score ranges from 0 to 10 to reflect the full response range of the scale. Data are displayed as mean and 95% confidence intervals. ADLs: activities of daily living; CKD: chronic kidney disease; KTR: kidney transplant recipients; MSS: muscle symptom scale; NDD: non-dialysis dependent. *Statistical difference to non-CKD; ^#^Statistical difference to non-dialysis dependent; ^§^Statistical difference to dialysis.

The impact of symptoms of muscle dysfunction over six months on ADLs are shown in Figure 3 panels: a) performing daily activities; b) socializing; c) working; d) exercising). For all ADLs, participants without CKD experienced significantly less impact from muscle symptoms compared to those with CKD (*p*-values < 0.05). The impact of these symptoms on ADLs was significantly worse for people on dialysis compared to people with NDD-CKD (*p*-values *<* 0.05). For KTR, the impact of symptoms of muscle dysfunction on ADLs was reduced compared to dialysis patients (*p*-values *<* 0.05), returning to similar levels as the NDD population.

### Association between symptoms of muscle dysfunction and impact on ADLs

Univariate associations between symptoms of muscle dysfunction and impact on ADLs in non-CKD and individuals with CKD are shown in Table S2. There were significant associations between all symptoms and their impact on ADLs, with increased symptomatology associating with reduced ability to perform ADLs. These relationships were most pronounced for weakness and reduced muscle size. When CKD is further stratified into subgroups, the univariate associations between each symptom and their impact on ADLs remained significant in all groups (Table S4). Muscle weakness consistently demonstrated the strongest association with reduced participation in ADLs across all groups. Tiredness was an important contributor to reduced ADLs in patients on dialysis and KTRs as was a reduction in muscle size in NDD-CKD and dialysis.

The pattern of multivariable associations differed between CKD and non-CKD participants (Table S3). In the CKD group, three symptoms, muscle weakness, perceived reduction in muscle size, and RLS, remained consistently associated with an impact upon all four domains, performing daily activities, socializing, working and exercising, indicating broad functional impact (all *p*-values < 0.05). Ache/pain was associated only with limitations in daily activities, and cramp/tightness only with socializing. In contrast, the non-CKD group demonstrated a different profile. Weakness remained associated with limitations in daily activities, exercise and working, and ache/pain showed wider associations with daily activities, socializing and working. Perceived reduction in muscle size was associated only with daily activities and socializing. Restless leg syndrome showed no association with any domain. These contrasting patterns suggest that while weakness and perceived muscle size contribute to functional limitations in both groups, RLS appears to be a CKD-specific determinant of impaired ADL’s, whereas ache/pain plays a more prominent role in non-CKD individuals.

In multivariable models stratified by CKD subgroup (Table 2), distinct symptom patterns were observed. In NDD-CKD, weakness and muscle size reduction were the strongest independent predictors across all four domains of ADL’s. Ache/pain was associated with reduced daily activities and reduced socializing. Cramp/tightness was associated with reduced socializing and working. In dialysis patients, muscle size reduction and RLS were consistently associated with reductions in all four ADL’s. No other symptoms were independently associated. Among KTR, weakness remained independently associated with daily activities and work participation, RLS associated with daily activities and work, and tiredness associated with exercise participation.

**Table 2.** Multivariable linear regression analysis for the association between symptoms of muscle dysfunction and impacts on activities of daily living in non-CKD and CKD subgroups.

Symptoms of muscle dysfunction	Impact on participation in ADLs							
	Daily activities		Socializing		Working		Exercising	
	*B* (95% CI)	*r* ^2^	*B* (95% CI)	*r* ^2^	*B* (95% CI)	*r* ^2^	*B* (95% CI)	*r* ^2^
Non-CKD								
Weakness	0.24 (0.09, 0.39)*	0.35	0.16 (−0.01, 0.31)	0.25	0.22 (0.03, 0.40)*	0.19	0.27 (0.12, 0.43)*	0.31
Tiredness	−0.01 (−0.14, 0.12)		0.10 (−0.04, 0.24)		−0.02 (−0.19, 0.15)		0.05 (−0.09, 0.19)	
Ache/pain	0.19 (0.06, 0.33)*		0.16 (0.02, 0.31)*		0.22 (0.05, 0.39)*		0.13 (−0.10, 0.27)	
Cramp/tightness	0.04 (−0.07, 0.15)		−0.01 (−0.13, 0.12)		0.01 (−0.14, 0.14)		0.10 (−0.02, 0.22)	
Reduction in size	0.26 (0.10, 0.42)*		0.19 (0.02, 0.35)*		0.08 (−0.11, 0.28)		0.07 (−0.09, 0.24)	
Restless leg syndrome	0.01 (−0.08, 0.11)		0.02 (−0.08, 0.13)		0.04 (−0.08, 0.16)		0.01 (−0.10, 0.10)	
NDD								
Weakness	0.32 (0.15, 0.50)*	0.53	0.22 (0.05, 0.39)*	0.42	0.27 (0.07, 0.47)*	0.30	0.41 (0.19, 0.63)*	0.34
Tiredness	0.06 (−0.08, 0.21)		0.02 (−0.12, 0.16)		0.07 (−0.10, 0.24)		0.04 (−0.14, 0.22)	
Ache/pain	0.32 (0.20, 0.45)*		0.14 (0.02, 0.27)*		0.03 (−0.12, 0.17)		0.08 (−0.07, 0.24)	
Cramp/tightness	0.03 (−0.07, 0.14)		0.18 (0.07, 0.28)*		0.16 (0.03, 0.29)*		0.09 (−0.05, 0.22)	
Reduction in size	0.22 (0.09, 0.36)*		0.25 (0.12, 0.38)*		0.17 (0.01, 0.33)*		0.19 (0.02, 0.36)*	
Restless leg syndrome	−0.01 (−0.11, 0.09)		0.02 (−0.07, 0.12)		−0.01 (−0.12, 0.11)		0.04 (−0.08, 0.16)	
Dialysis								
Weakness	0.18 (−0.02, 0.38)	0.49	0.10 (−0.15, 0.34)	0.31	0.16 (−0.12, 0.43)	0.30	0.09 (−0.18, 0.36)	0.28
Tiredness	0.12 (−0.08, 0.32)		0.04 (−0.21, 0.29)		0.13 (−0.14, 0.40)		0.25 (−0.02, 0.52)	
Ache/pain	0.09 (−0.09, 0.26)		0.12 (−0.09, 0.33)		0.10 (−0.14, 0.33)		0.09 (−0.14, 0.32)	
Cramp/tightness	0.03 (−0.12, 0.18)		0.12 (−0.07, 0.31)		−0.01 (−0.22, 0.21)		−0.11 (−0.32, 0.11)	
Reduction in size	0.26 (0.14, 0.37)*		0.29 (0.15, 0.43)*		0.32 (0.17, 0.48)*		0.32 (0.16, 0.47)*	
Restless leg syndrome	0.28 (0.17, 0.39)*		0.16 (0.02, 0.30)*		0.15 (−0.01, 0.30)*		0.16 (0.01, 0.31)*	
KTR								
Weakness	0.34 (0.07, 0.61)*	0.54	0.28 (−0.03, 0.58)	0.37	0.38 (0.06, 0.71)*	0.40	0.20 (−0.12, 0.52)	0.45
Tiredness	0.17 (−0.13, 0.46)		0.10 (−0.23, 0.42)		0.32 (−0.02, 0.66)		0.40 (0.05, 0.75)*	
Ache/pain	0.06 (−0.19, 0.31)		−0.11 (−0.39, 0.17)		−0.14 (−0.43, 0.15)		−0.13 (−0.43, 0.17)	
Cramp/tightness	0.04 (−0.17, 0.25)		0.02 (−0.21, 0.25)		−0.02 (−0.27, 0.22)		0.06 (−0.19, 0.31)	
Reduction in size	0.05 (−0.14, 0.24)		0.20 (−0.01, 0.41)		−0.11 (−0.33, 0.11)		0.17 (−0.05, 0.40)	
Restless leg syndrome	0.26 (0.10, 0.43)*		0.18 (−0.01, 0.36)		0.26 (0.07, 0.45)*		0.18 (−0.01, 0.38)	

ADLs: activities of daily living; CKD: chronic kidney disease.

Models adjusted for age, sex, and diabetic status.

*Indicates a significant association (*p*-value < 0.05).

## Discussion

Symptom burden is high in people with CKD, and our findings here show that muscle related symptoms are more severe in individuals with CKD compared to those without the disease. Within the CKD spectrum, muscle symptom severity increased with disease progression; it was greatest in those receiving dialysis, and remained elevated in KTR compared to NDD-CKD. These symptoms were also strongly linked to greater limitations in ADLs, particularly in dialysis patients, with transplant recipients showing partial improvement. To our knowledge, this is the first study to compare muscle symptoms and their functional impacts across the full CKD continuum alongside a non-CKD cohort.

Loss of muscle mass, strength, and physical function is well documented in CKD, and is associated with increased morbidity and mortality [18]. From the patient perspective, it can severely impair their ability to perform meaningful activities and maintain independence, directly impacting quality of life. Despite burden, muscle-related symptoms are often under-recognized and inadequately managed in routine nephrology care.

Our results align with previous work showing that musculoskeletal symptoms are common in CKD [8], and particularly pronounced in the dialysis population [19] where prevalence increases with dialysis vintage and age [20]. In this study, patient-reported symptoms, including muscle weakness, tiredness, aches and pains, cramps, atrophy and RLS were more frequent in NDD-CKD, dialysis, and KTR, compared to individuals without CKD, with the highest burden observed in those receiving dialysis.

Consistent with our findings, muscle weakness and fatigue has previously been identified as the most common symptom across all stages of CKD often clustering with other symptoms such as poor appetite, depression, anxiety and poor mobility [21]. These symptoms frequently coexist with muscle wasting, further compounding their impact on physical function and quality of life. Restless leg syndrome is also significantly more prevalent in the CKD population, occurring at rates 2–3 times higher than in the general population [22], with up to 25% of individuals on dialysis reporting the symptom [23]. Muscle cramps are another common complaint across the CKD spectrum, but are particularly prevalent in patients receiving dialysis, likely due to fluid and electrolyte imbalances [24]. Additionally, a recent systematic review of 13 studies identified muscle and joint pain as the most frequently reported symptom, with prevalence estimates ranging from 25% to 83% across different stages of CKD, underscoring the high and variable burden of musculoskeletal symptoms in this population.

It was interesting to note the different contributions of the muscle symptoms to limitations in ADL’s between CKD and non-CKD individuals. In CKD, weakness, perceived muscle size loss, and RLS were associated with limitations within all ADL domains. In contrast, ache/pain played a larger role in non-CKD participants, while showing only limited associations in CKD. The lack of ADL associations for RLS in non-CKD, highlights the possibility of CKD-specific mechanisms shaping how symptoms affect daily functioning.

Furthermore, stratification by CKD subgroup revealed that these associations were not uniform across the CKD spectrum and were instead driven by distinct symptom profiles at different stages of disease and treatment. In NDD-CKD, the strong associations observed in the grouped CKD analysis were primarily driven by consistent and independent effects of muscle weakness and muscle size reduction across all ADL domains. In contrast, among dialysis patients, weakness and tiredness no longer retained independent associations after adjustment, and the overall CKD effects for perceived muscle size reduction and RLS were largely attributable to this subgroup. Specifically, muscle size reduction and RLS emerged as the dominant independent predictors of impaired daily activities, social participation, work, and exercise in dialysis patients. Among KTR, the persistence of significant associations for weakness and RLS in the grouped CKD analysis was driven by domain-specific effects, particularly for daily activities and working. Unlike the other groups, not one symptom consistently impacted all ADL’s in KTR, which could indicate the aforementioned ease of burden in this group. These findings highlight that aggregating CKD stages may obscure clinically meaningful differences in the mechanisms underlying functional limitation, and underscore the importance of stratified analyses to accurately characterize symptom-specific drivers of disability across the CKD trajectory.

The partial improvement observed in transplant recipients likely reflects the partial reversal of the uremic milieu. Longitudinal studies show that muscle mass and strength generally improve after kidney transplantation, but often remain subnormal, and sarcopenia remains common in this population [25]. Persistent risk factors such as low physical activity, nutritional deficiencies, hyperparathyroidism and other metabolic complications have all been implicated in post-transplant sarcopenia and impaired muscle function [26]. Immunosuppressive regimens can also contribute to sarcopenia as suppression and dysregulation of immune pathways can impair muscle repair, alter inflammatory signally and negatively influence protein turnover [27].

From a clinical perspective, these findings highlight symptoms, particularly weakness, perceived muscle size loss, and restless legs, that may be amenable to targeted intervention. Exercise-based programmes have consistently been shown to improve strength, physical function and symptom burden in CKD [28–31], and our results suggest that individuals with more severe symptoms profiles may derive particular benefit. Nutritional strategies aimed at supporting muscle mass may also be relevant, especially for patients reporting perceived size loss. Integrating symptom monitoring into routine care, possibly using patient reported outcome measures (PROMS), could help identify patients most likely to respond to exercise or nutritional interventions and guide the timing and personalization of rehabilitation approaches across CKD stages.

The underlying causes of these symptoms remain poorly understood, which has hindered the development of targeted and effective treatments. However, growing evidence suggests that many of these muscle-related symptoms are responsive to non-pharmacological interventions, particularly exercise [32,33]. Structured exercise programmes have been shown to improve muscle strength, physical function, and fatigue in individuals with CKD [28,29], highlighting their potential as a practical and impactful therapeutic strategy.

Qualitative data on muscle specific symptoms in people with CKD is lacking. Such insights are critical to deepening our understanding of how these symptoms impact patients’ daily lives and identifying the most appropriate timing and context for interventions, such as exercise, targeted toward those most likely to benefit. Future clinical trials targeting muscle dysfunction should capture and assess responses to symptoms, which can be monitored by patients themselves. By adopting these measures as PROMs [34], patients may be motivated to adhere to lifestyle changes, such as exercise, activity and diet.

A key strength of the I-RACE study lies in its large, well-characterized cohort of individuals living with and without CKD, spanning all stages of the disease and recruited from multiple sites across the UK. Despite this there are several limitations that should be acknowledged. First the cross-sectional design precludes causal inference, and the associations observed between muscle symptoms and ADL limitations should be interpreted as correlational. Secondly, while we were able to capture information on kidney function and disease stage, we did not assess additional indicators that reflect the broader risk of CKD progression, cardiovascular disease, and other adverse health outcomes. It is possible that muscle-related symptoms, limitations in ADLs, and their interaction, are influenced not only by the current stage of kidney disease but also by underlying risk factors for disease progression and complications. Third, although we adjusted for key demographic and clinical variables (age, sex and diabetes), we were unable to adjust for potentially relevant covariates such as BMI, nutritional status, inflammatory markers, or dialysis duration as these data were not collected. This may have resulted in residual confounding. Fourth, the MSS used in this study was specifically developed to capture the most commonly reported muscle-related symptoms in people living with CKD (e.g. cramps, restless legs), which are often overlooked by existing instruments. The MSS has not undergone formal psychometric validation, and thus interpretation and generalizability of the findings should be approached with caution. Comparisons with studies using other validated tools may also be limited. Future work should perform formal psychometric validation of the MSS to support its wider use in CKD research and clinical practice.

## Conclusion

Symptoms of muscle dysfunction were more severe across the spectrum of CKD, with the greatest burden observed in dialysis patients and partial improvement seen in kidney transplant recipients. Among these symptoms, muscle weakness in NDD-CKD and perceived reduction in muscle size in NDD-CKD and dialysis were most strongly associated with limitations in ADLs. These findings highlight the importance of recognizing and addressing muscle-related symptoms in routine clinical care. A multidisciplinary approach that targets these symptoms may help preserve functional independence and quality of life in people living with CKD. Future interventional trials should prioritize muscle weakness and muscle size as modifiable predictors of impaired daily functioning.

## Supplementary Material

Supplementary Table S1.docx

Supplementary Table S4.docx

Supplementary material_1.docx

Supplementary Table S2.docx

Supplementary Table S3.docx

## Data Availability

Data are available from the corresponding author upon reasonable request.
