# Supplementary material for: Muscle weakness, pain, and fatigue impair daily function in chronic kidney disease: a cross-sectional analysis from the I-RACE study
Source: Ren Fail. 2026 Mar 4;48(1):2637300. doi: 10.1080/0886022X.2026.2637300 (PMC12964459; doi:10.1080/0886022X.2026.2637300)
Supplement: Supplementary Table S1.docx [file IRNF_A_2637300_SM0220.docx]

**Supplementary Table S1.** Numerical values of the MSS score according to the CKD status.

| **MSS** | **Non-CKD**  **(n = 304)** | **NDD-CKD**  **(n = 345)** | **Dialysis**  **(n = 281)** | **KTR**  **(n = 118)** |
| --- | --- | --- | --- | --- |
| **Symptoms of muscle dysfunction** | |  |  |  |
| Weakness | 1.9 (1.6 – 2.3) | 3.8 (3.5 – 4.2) | 5.5 (5.2 – 5.9) | 4.2 (3.6 – 4.8) |
| Tiredness | 2.5 (2.2 – 2.8) | 4.2 (3.9 – 4.6) | 5.6 (5.2 – 6.0) | 5.1 (4.4 – 5.7) |
| Pain/Aches | 2.7 (2.4 – 3.0) | 4.3 (3.9 – 4.7) | 5.4 (5.0 – 5.8) | 4.7 (4.1 – 5.3) |
| Cramp/tightness | 2.4 (2.1 – 2.7) | 4.0 (3.6 – 4.4) | 5.2 (4.8 – 5.6) | 4.5 (3.8 – 5.1) |
| **Symptoms of muscle dysfunction over the past 6 months** | | |  |  |
| Reduction in muscle size | 0.7 (0.5 – 0.9) | 2.4 (2.1 – 2.8) | 4.7 (4.2 – 5.1) | 3.5 (2.9 – 4.2) |
| Restless leg syndrome | 1.6 (1.3 – 1.9) | 2.7 (2.3 – 3.1) | 3.8 (3.4 – 4.3) | 3.7 (3.0 – 4.5) |
| **Impact of symptoms on ADLs** | |  |  |  |
| Performing daily activities | 1.1 (0.9 – 1.4) | 3.0 (2.6 – 3.4) | 5.2 (4.8 – 5.6) | 3.4 (2.7 – 4.0) |
| Socialising | 1.0 (0.8 – 1.3) | 2.3 (1.9 – 2.7) | 4.8 (4.3 – 5.3) | 2.8 (2.1 – 3.4) |
| Working | 1.1 (0.8 – 1.4) | 2.1 (1.7 – 2.5) | 4.5 (4.0 – 5.0) | 2.6 (1.9 – 3.3) |
| Exercising | 1.3 (1.0 – 1.5) | 3.2 (2.7 – 3.6) | 4.9 (4.4 – 5.4) | 3.6 (2.9 – 4.3) |
| **Total MSS score** | 15.9 (13.8 – 17.9) | 31.0 (28.3 – 33.7) | 48.4 (45.2 – 51.6) | 36.4 (31.7 – 41.1) |

ADLs, activities of daily living; CVD, cardiovascular disease; KTR, kidney transplant recipients; MSS, muscle symptom scale; NDD, non-dialysis dependent.

Data are displayed as mean and 95% confidence intervals.
