# Supplementary material for: Muscle weakness, pain, and fatigue impair daily function in chronic kidney disease: a cross-sectional analysis from the I-RACE study
Source: Ren Fail. 2026 Mar 4;48(1):2637300. doi: 10.1080/0886022X.2026.2637300 (PMC12964459; doi:10.1080/0886022X.2026.2637300)
Supplement: Supplementary Table S4.docx [file IRNF_A_2637300_SM0219.docx]

**Supplementary Table S4. Univariate linear regression analysis for the association between symptoms of muscle dysfunction and impacts on activities of daily living in non-CKD and CKD subgroups.**

| **Symptoms of muscle dysfunction** | **Impact on participation in ADLs** | | | | | | | |
| --- | --- | --- | --- | --- | --- | --- | --- | --- |
|  | **Daily activities** | | **Socialising** | | **Working** | | **Exercising** | |
|  | ***B* (95% CI)** | **r^2^** | ***B* (95% CI)** | **r^2^** | ***B* (95% CI)** | **r^2^** | ***B* (95% CI)** | **r^2^** |
| **Non-CKD** |  |  |  |  |  |  |  |  |
| Weakness | 0.49 (0.39, 0.55)* | 0.26 | 0.41 (0.31, 0.51)* | 0.19 | 0.40 (0.28, 0.51)* | 0.13 | 0.48 (0.38, 0.58)* | 0.23 |
| Tiredness | 0.37 (0.27, 0.47)* | 0.16 | 0.36 (0.26, 0.45)* | 0.15 | 0.29 (0.18, 0.41)* | 0.08 | 0.41 (0.32, 0.50)* | 0.22 |
| Ache/pain | 0.41 (0.33, 0.50)* | 0.24 | 0.35 (0.26, 0.44)* | 0.17 | 0.25 (0.14, 0.36)* | 0.06 | 0.41 (0.32, 0.50)* | 0.23 |
| Cramp/tightness | 0.33 (0.24, 0.43)* | 0.14 | 0.26 (0.16, 0.36)* | 0.09 | 0.27 (0.15, 0.38)* | 0.07 | 0.34 (0.24, 0.44)* | 0.14 |
| Reduction in size | 0.58 (0.44, 0.72)* | 0.18 | 0.47 (0.33, 0.62)* | 0.12 | 0.38 (0.21, 0.56)* | 0.06 | 0.46 (0.31, 0.61)* | 0.10 |
| Restless leg syndrome | 0.21 (0.11, 0.31)* | 0.05 | 0.18 (0.08, 0.28)* | 0.04 | 0.16 (0.04, 0.27)* | 0.02 | 0.18 (0.08, 0.29)* | 0.04 |
| **NDD** |  |  |  |  |  |  |  |  |
| Weakness | 0.75 (0.66, 0.84)* | 0.45 | 0.57 (0.47, 0.67)* | 0.32 | 0.52 (0.41, 0.63)* | 0.23 | 0.66 (0.54, 0.77)* | 0.31 |
| Tiredness | 0.61 (0.51, 0.71)* | 0.33 | 0.46 (0.37, 0.56)* | 0.24 | 0.44 (0.33, 0.54)* | 0.19 | 0.52 (0.40, 0.63)* | 0.21 |
| Ache/pain | 0.62 (0.53, 0.71)* | 0.37 | 0.46 (0.36, 0.55)* | 0.25 | 0.37 (0.26, 0.47)* | 0.14 | 0.45 (0.33, 0.56)* | 0.17 |
| Cramp/tightness | 0.45 (0.34, 0.56)* | 0.19 | 0.45 (0.35, 0.55)* | 0.22 | 0.39 (0.28, 0.50)* | 0.15 | 0.40 (0.28, 0.52)* | 0.13 |
| Reduction in size | 0.63 (0.51, 0.75)* | 0.27 | 0.55 (0.44, 0.66)* | 0.25 | 0.46 (0.34, 0.58)* | 0.16 | 0.57 (0.44, 0.70)* | 0.20 |
| Restless leg syndrome | 0.27 (0.15, 0.40)* | 0.07 | 0.25 (0.14, 0.36)* | 0.07 | 0.18 (0.06, 0.30)* | 0.03 | 0.25 (0.12, 0.38)* | 0.05 |
| **Dialysis** |  |  |  |  |  |  |  |  |
| Weakness | 0.68 (0.56, 0.79)* | 0.34 | 0.54 (0.41, 0.68)* | 0.20 | 0.57 (0.42, 0.72)* | 0.18 | 0.54 (0.39, 0.68)* | 0.17 |
| Tiredness | 0.63 (0.52, 0.74)* | 0.32 | 0.51 (0.38, 0.64)* | 0.19 | 0.54 (0.39, 0.68)* | 0.18 | 0.54 (0.40, 0.68)* | 0.18 |
| Ache/pain | 0.57 (0.46, 0.68)* | 0.28 | 0.50 (0.37, 0.63)* | 0.19 | 0.50 (0.36, 0.64)* | 0.16 | 0.46 (0.32, 0.60)* | 0.14 |
| Cramp/tightness | 0.54 (0.43, 0.66)* | 0.24 | 0.50 (0.37, 0.63)* | 0.19 | 0.48 (0.33, 0.63)* | 0.14 | 0.41 (0.26, 0.55)* | 0.11 |
| Reduction in size | 0.55 (0.44, 0.66)* | 0.27 | 0.51 (0.39, 0.63)* | 0.21 | 0.56 (0.42, 0.69)* | 0.21 | 0.53 (0.39, 0.66)* | 0.19 |
| Restless leg syndrome | 0.56 (0.46, 0.67)* | 0.29 | 0.43 (0.31, 0.56)* | 0.16 | 0.42 (0.28, 0.56)* | 0.13 | 0.40 (0.27, 0.54)* | 0.12 |
| **KTR** |  |  |  |  |  |  |  |  |
| Weakness | 0.74 (0.58, 0.90)* | 0.43 | 0.52 (0.34, 0.70)* | 0.25 | 0.59 (0.40, 0.78)* | 0.29 | 0.68 (0.49, 0.88)* | 0.32 |
| Tiredness | 0.63 (0.47, 0.79)* | 0.38 | 0.44 (0.27, 0.62)* | 0.21 | 0.49 (0.31, 0.68)* | 0.23 | 0.67 (0.49, 0.85)* | 0.36 |
| Ache/pain | 0.54 (0.37, 0.71)* | 0.28 | 0.33 (0.15, 0.51)* | 0.12 | 0.35 (0.16, 0.54)* | 0.12 | 0.46 (0.26, 0.66)* | 0.18 |
| Cramp/tightness | 0.52 (0.33, 0.70)* | 0.23 | 0.34 (0.16, 0.53)* | 0.12 | 0.35 (0.15, 0.55)* | 0.11 | 0.47 (0.26, 0.68)* | 0.16 |
| Reduction in size | 0.45 (0.27, 0.63)* | 0.20 | 0.43 (0.26, 0.60)* | 0.21 | 0.35 (0.17, 0.54)* | 0.13 | 0.52 (0.32, 0.71)* | 0.23 |
| Restless leg syndrome | 0.47 (0.32, 0.63)* | 0.26 | 0.35 (0.20, 0.51)* | 0.17 | 0.40 (0.23, 0.57)* | 0.19 | 0.41 (0.23, 0.59)* | 0.17 |

Abbreviations: CKD, chronic kidney disease; KTR, kidney transplant recipients; MSS, muscle symptom scale; NDD, non-dialysis dependent.

* indicates a significant association (*p*-value < 0.05).
