# Supplementary material for: Muscle weakness, pain, and fatigue impair daily function in chronic kidney disease: a cross-sectional analysis from the I-RACE study
Source: Ren Fail. 2026 Mar 4;48(1):2637300. doi: 10.1080/0886022X.2026.2637300 (PMC12964459; doi:10.1080/0886022X.2026.2637300)

**SUPPLEMENTARY MATERIAL**

**Supplementary material 1:** Leicester Kidney Exercise Team muscle symptom scale


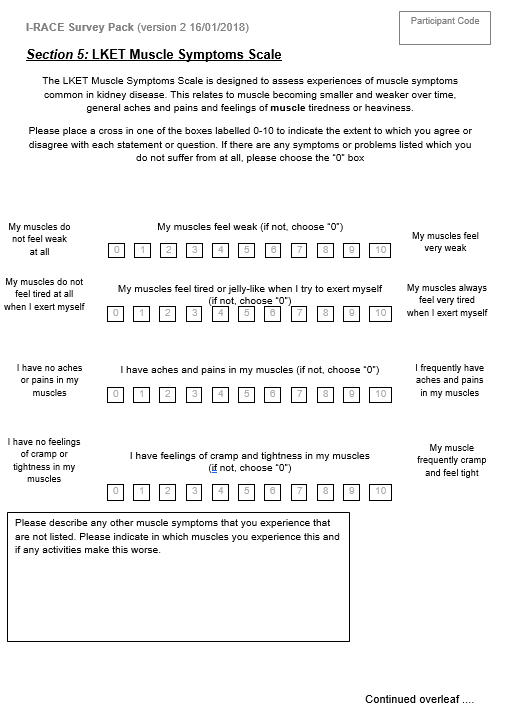


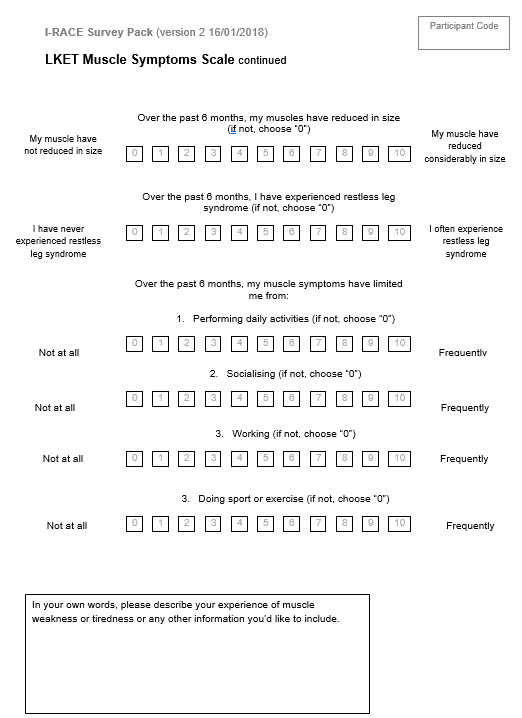

Supplement: Supplementary material_1.docx [file IRNF_A_2637300_SM0218.docx]
