# Supplementary material for: Muscle weakness, pain, and fatigue impair daily function in chronic kidney disease: a cross-sectional analysis from the I-RACE study
Source: Ren Fail. 2026 Mar 4;48(1):2637300. doi: 10.1080/0886022X.2026.2637300 (PMC12964459; doi:10.1080/0886022X.2026.2637300)
Supplement: Supplementary Table S3.docx [file IRNF_A_2637300_SM0216.docx]

**Supplementary Table S3. Multivariable linear regression analysis for the association between symptoms of muscle dysfunction and impacts on activities of daily living in non-CKD and CKD individuals.**

| **Symptoms of muscle dysfunction** | **Impact on participation in ADLs** | | | | | | | |
| --- | --- | --- | --- | --- | --- | --- | --- | --- |
|  | **Daily activities** |  | **Socialising** |  | **Working** |  | **Exercising** |  |
|  | **B (95% CI)** | **r^2^** | **B (95% CI)** | **r^2^** | **B (95% CI)** | **r^2^** | **B (95% CI)** | **r^2^** |
| **Non-CKD** |  |  |  |  |  |  |  |  |
| Weakness | 0.24 (0.09, 0.39)* |  | 0.16 (-0.01, 0.31) |  | 0.22 (0.03, 0.40)* |  | 0.27 (0.12, 0.43)* |  |
| Tiredness | -0.01 (-0.14, 0.12) |  | 0.10 (-0.04, 0.24) |  | -0.02 (-0.19, 0.15) |  | 0.05 (-0.09, 0.19) |  |
| Ache/pain | 0.19 (0.06, 0.33)* | 0.35 | 0.16 (0.02, 0.31)* | 0.25 | 0.22 (0.05, 0.39)* | 0.19 | 0.13 (-0.10, 0.27) | 0.31 |
| Cramp/tightness | 0.04 (-0.07, 0.15) |  | -0.01 (-0.13, 0.12) |  | 0.01 (-0.14, 0.14) |  | 0.10 (-0.02, 0.22) |  |
| Reduction in size | 0.26 (0.10, 0.42)* |  | 0.19 (0.02, 0.35)* |  | 0.08 (-0.11, 0.28) |  | 0.07 (-0.09, 0.24) |  |
| Restless leg syndrome | 0.01 (-0.08, 0.11) |  | 0.02 (-0.08, 0.13) |  | 0.04 (-0.08, 0.16) |  | 0.01 (-0.10, 0.10) |  |
| **CKD** |  |  |  |  |  |  |  |  |
| Weakness | 0.34 (0.22, 0.46)* |  | 0.25 (0.12, 0.38)* |  | 0.30 (0.15, 0.44)* |  | 0.28 (0.13, 0.42)* |  |
| Tiredness | 0.07 (-0.40, 0.18) |  | 0.01 (-0.11, 0.13) |  | 0.08 (-0.05, 0.22) |  | 0.13 (-0.01, 0.27) |  |
| Ache/pain | 0.18 (0.09, 0.28)* | 0.52 | 0.08 (-0.02, 0.18) | 0.40 | 0.01 (-0.10, 0.13) | 0.33 | 0.06 (-0.06, 0.18) | 0.34 |
| Cramp/tightness | 0.04 (-0.04, 0.12) |  | 0.14 (0.05, 0.24)* |  | 0.10 (-0.01, 0.20) |  | 0.03 (-0.07, 0.14) |  |
| Reduction in size | 0.23 (0.15, 0.31)* |  | 0.29 (0.21, 0.38)* |  | 0.23 (0.13, 0.33)* |  | 0.26 (0.16, 0.36)* |  |
| Restless leg syndrome | 0.14 (0.07, 0.20)* |  | 0.09 (0.02, 0.16)* |  | 0.09 (0.01, 0.17)* |  | 0.09 (0.01, 0.17)* |  |

Abbreviations: ADLs, activities of daily living; CKD, chronic kidney disease.

Models adjusted for age, sex, and diabetic status.

* indicates a significant association (*p*-value < 0.05).
